# The complete chloroplast genome sequence of *Olea dioica* Roxb, 1820 (Oleaceae)

**DOI:** 10.1080/23802359.2024.2366373

**Published:** 2024-06-17

**Authors:** Jinhua Long, Yang Tian, Jianguo Zhang, Zhaoshan Wang

**Affiliations:** a Key Laboratory of Silviculture of the State Forestry Administration, Research Institute of Forestry, Chinese Academy of Forestry, Beijing, China; b Collaborative Innovation Center of Sustainable Forestry in Southern China, Nanjing Forestry University, Nanjing, China

**Keywords:** Complete chloroplast genome, *Olea dioica*, Oleaceae, phylogenetic analysis

## Abstract

*Olea dioica* Roxb, 1820 is a very important ethnomedicinal tree because of its medicinal properties and it belongs to the Oleaceae family. It is mainly distributed in evergreen and semi-evergreen forests. However, the chloroplast genome of *O. dioica* has not yet been reported. In this study, the chloroplast genome sequence of *O. dioica* was sequenced using next-generation sequencing technologies. The complete chloroplast genome of *O. dioica* was 155,138 bp in length (GenBank accession no. PP048999), comprising a large single-copy (LSC) region of 86,048 bp, a small single-copy (SSC) region of 17,816 bp, and two inverted repeat (IR) regions 25,637 bp each. The overall GC content was 37.8%. The complete chloroplast genome of *O. dioica* contains 131 complete genes, which are 88 protein-coding genes, 35 transfer RNA genes, and eight ribosomal RNA genes. A maximum-likelihood (ML) tree of *O. dioica* and 14 other species in the family Oleaceae suggested that *O. dioica* showed a close relationship with *Olea brachiata*.

## Introduction

**Figure 1.**
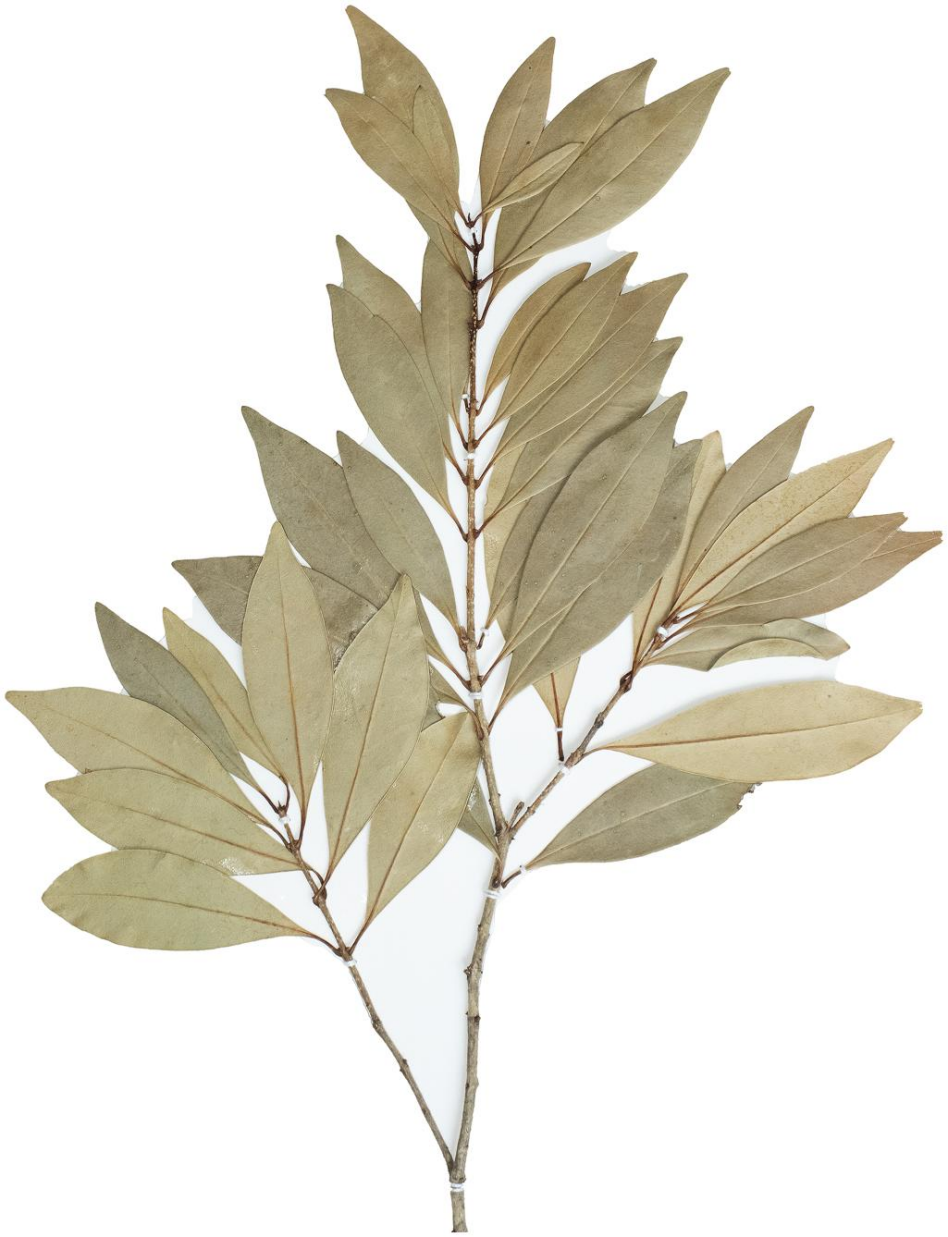
Morphological characteristics of *Olea dioica*. The photo was taken by the author Zhaoshan Wang at the Research Institute of Forestry, Chinese Academy of Forestry. The leaves of *O. dioica* are blade leathery, lanceolate, oblanceolate or long elliptic-lanceolate, apex acuminate or obtuse, sparsely rounded, base cuneate, entire or irregularly and sparsely serrate, usually reddish when dry.

*Olea dioica* Roxb, 1820 is an important medicinal tree plant belonging to the Oleaceae family. It was described by Roxb in 1820 (Green 2003). It is being cultivated in Guangdong, Guangxi, southeastern Guizhou, and southern Yunnan, China, and is commonly found in the Western Ghats, India. The tree grows up to 15 m tall, the bark of the tree is gray, grayish-white or gray branches, the leaves are blade leathery, lanceolate, oblanceolate or long elliptic-lanceolate, 5–10 cm long, 1.5–3.7 cm wide, apex acuminate or obtuse, sparsely rounded, base cuneate, entire or irregularly and sparsely serrate, usually reddish when dry, cymes paniculate, sometimes racemose or umbellate, inflorescence axillary, flowers polygamodioecious, cream-white, fruit ellipsoid or ovate, black or purple-black at maturity (Figure 1). In previous studies, *O. dioica* has been identified as a valuable medicinal plant due to its various pharmacological activities. In Siddha medicine, the roots of this plant are used to treat cancer and snake bites. In Maharashtra, tribes use the fruits of *O. dioica* to treat skin diseases. The bark and fruit paste are used in rheumatism; decoction of the bark is used to wash old wounds and is given to counter fever (Pullaiah 2006). Ripe fruits are traditionally used in various treatments by the tribes in the Kerala forest (Yesodharan and Sujana 2007). Despite its numerous potentialities, such as significant antioxidant, antibacterial, antifungal, anticholinesterase, aphrodisiac, cytotoxic, and analgesic activity (Poornima et al. 2012; Kekuda and Raghavendra 2014; Ashwathanarayana and Naika 2015a, 2015b, 2017a, 2017b; Pratap et al. 2020), this species continues to be neglected and underutilized. Here, we report the first complete chloroplast genome assembly of *O. dioica*, which will support further phylogeographic and population genetic studies of this species.

## Materials and methods

The fresh leaves of *O. dioica* were collected from Xinglong Town, Wanning City, Hainan Province, China (N 18°43′59″, E 110°11′41″). The voucher specimen (accession number: HCAF-OD0016; contact person: Jinhua Long; email: longjinhua97@gmail.com) was deposited in the herbarium of the Chinese Academy of Forestry. Total genomic DNA was isolated from silica-gel-dried leaves using the modified CTAB method (Doyle and Doyle 1987). Paired-end (PE) sequencing libraries with an insert size of 500 bp were constructed according to the Illumina library preparation protocol. Sequencing was performed on the Illumina HiSeq 2000 platform (Illumina, San Diego, CA), producing 150 bp PE reads. Adaptors were removed and low‐quality reads were filtered using Trimmomatic v0.39 (Bolger et al. 2014), and 57.33 Gb filtered reads of PE sequences were obtained; the clean data were assembled using the program GetOrganelle v1.7.7.0 (Jin et al. 2020) with k-mer set to 21, 77, and 127. Chloroplast annotation was performed on the online software CPGAVAS2 (http://47.96.249.172:16019/analyzer/home) (Shi et al. 2019) with the complete chloroplast genome of *O. europaea* subsp. *europaea* var. *europaea* (GenBank accession number: MG255763) serving as a reference and missing or incorrect genes were manually proofread in Geneious Prime 2022.2.1 (https://www.geneious.com) and GeSeq (https://chlorobox.mpimp-golm.mpg.de/geseq.html) (Tillich et al. 2017). The map of the chloroplast genome was drawn using the online tool CPGview (http://www.1kmpg.cn/cpgview/) (Liu et al. 2023). The coverage depth was generated using Burrow-Wheeler Aligner (BWA) (Li and Durbin 2009) by aligning sequencing data onto the chloroplast genome of *O. dioica*. Cis-splicing and trans-splicing genes were processed using CPGview (Liu et al. 2023). To analyze the phylogenetic position of *O. dioica*, the chloroplast genomes of 14 other species in the Oleaceae family were downloaded from the NCBI GenBank database. The sequence alignment of these 15 species was performed by using MAFFT v7.505 (Katoh and Standley 2013). The best nucleotide substitution model (GTR + G + I) was identified using MEGA11 (Tamura et al. 2021). The maximum-likelihood (ML) phylogenetic tree was then constructed using MEGA11 with 1000 bootstrap replicates with *Fraxinus velutina* treated as an outgroup.

## Results

**Figure 2.**
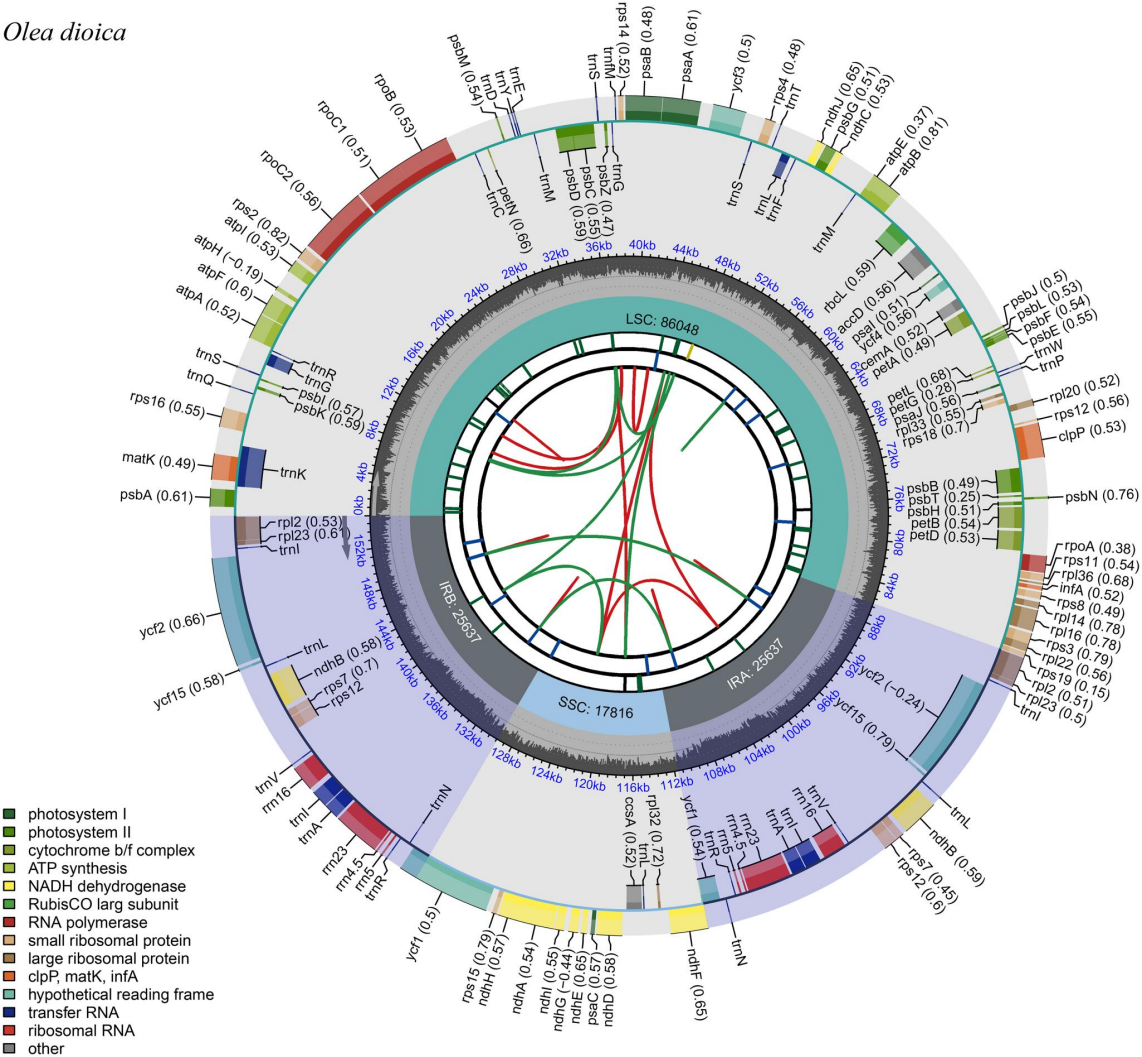
The circular map of the chloroplast genome of *Olea dioica*. Genes belonging to different functional groups are plotted in the outer circle. The functional classification of the genes is shown at the bottom left. The dark gray in the inner circle indicates the GC content of the chloroplast genome. The quadripartite structure, which consists of the LSC, the SSC, and two IR regions, is shown. The inner track shows the forward and reverse repeats connected with red and green arcs.

**Figure 3.**
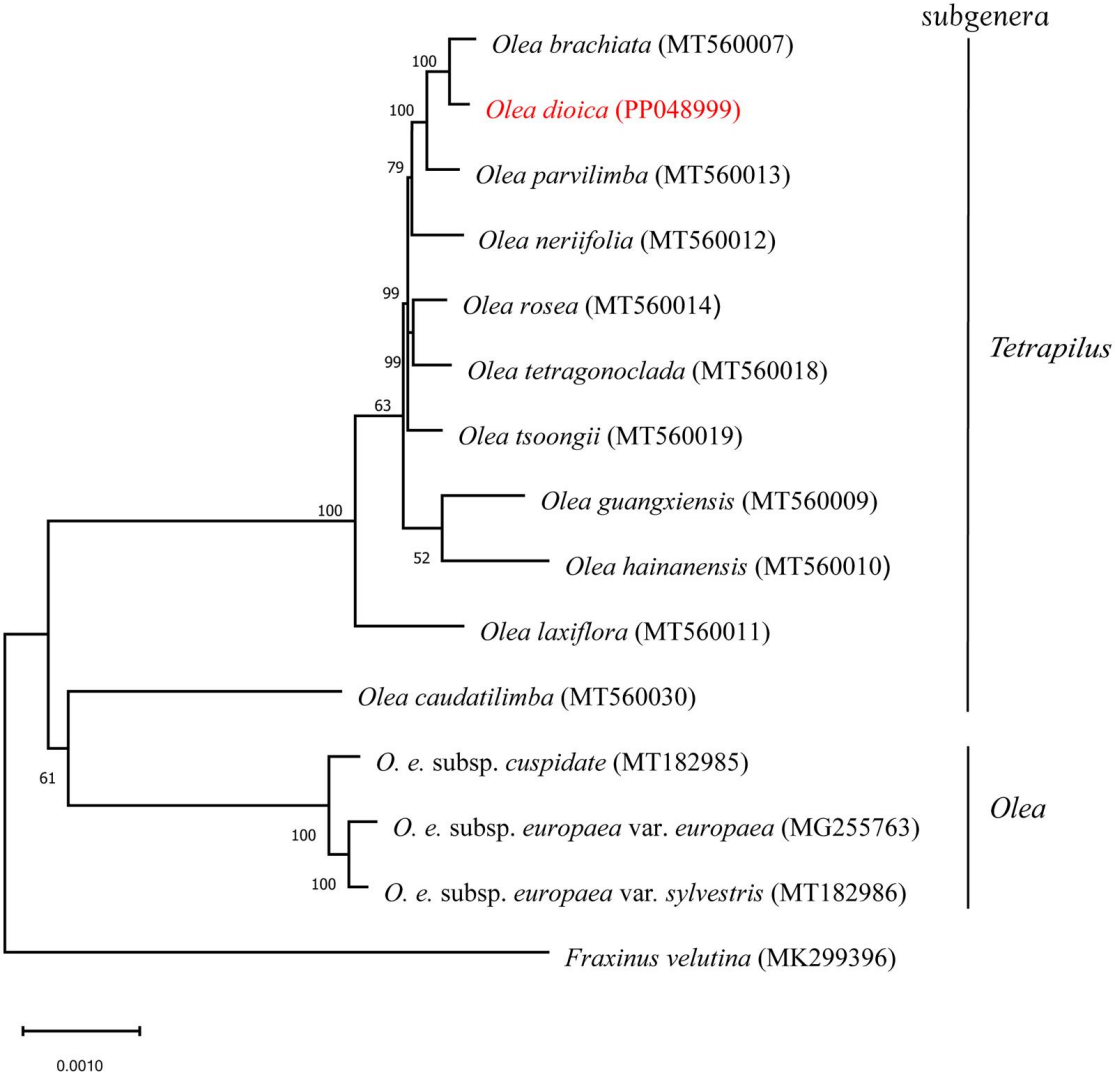
Maximum-likelihood (ML) phylogenetic tree with evolutionary distances for *Olea dioica* and 14 related taxa from the family Oleaceae. *Fraxinus velutina* is used as an outgroup. Numbers at the nodes indicated bootstrap support values from 1000 replicates. Accession numbers: *Olea brachiata*, MT560007 (Dong et al. 2022); *Olea dioica* (this study); *Olea parvilimba*, MT560013 (Dong et al. 2022); *Olea neriifolia*, MT560012 (Dong et al. 2022); *Olea rosea*, MT560014 (Dong et al. 2022); *Olea tetragonoclada*, MT560018 (Dong et al. 2022); *Olea tsoongii*, MT560019 (Dong et al. 2022); *Olea guangxiensis*, MT560009 (Dong et al. 2022); *Olea hainanensis*, MT560010 (Dong et al. 2022); *Olea laxiflora*, MT560011 (Dong et al. 2022); *Olea europaea* subsp. *cuspidata*, MT182985 (Niu et al. 2020); *Olea europaea* subsp. *europaea* var. *europaea*, MG255763 (reference unavailable); *Olea europaea* subsp. *europaea* var. *sylvestris*, MT182986 (Niu et al. 2020); *Olea caudatilimba*, MT560030 (Dong et al. 2022); *Fraxinus velutina*, MK299396 (Olofsson et al. 2019).

A total of 57.33 Gb cleaned reads of *O. dioica* were used for de novo chloroplast genome assembly by GetOrganelle and provided an average coverage depth of 7881.51× (Supplementary material, Figure S1). The complete chloroplast genome of *O. dioica* was 155,138 bp in length. The complete chloroplast genome sequence of this species has been deposited into the NCBI GenBank database under the accession number PP048999. The genome had a typical quadripartite structure, consisting of a large single-copy (LSC) region of 86,048 bp, two inverted repeat regions (IRs) of 25,637 bp each, and a small single-copy (SSC) region of 17,816 bp (Figure 2). The overall GC content was determined to be 37.8%, with the LSC exhibiting a corresponding value of 35.9%, the SSC at 31.9%, and the IR regions at 43.2%. A total of 131 complete genes were identified within the chloroplast genome of *O. dioica*: these included 88 protein-coding genes representing 80 unique species of protein-coding genes; as well as 35 transfer RNA (tRNA) genes encompassing a range of 26 distinct tRNA species; finally, there were also eight ribosomal RNA (rRNA) genes accounting for four different rRNA species. Among these genes, 14 cis-splicing genes, including *rps*16, *atp*F, *rpo*C1, *ycf*3, *clp*P, *pet*B, *pet*D, *rpl*16, and of *rpl*2 (two copies), *ndh*B (two copies), *ndh*A and *cyf*1 were discovered (Supplementary Figure S2). The trans-splicing gene *rps*12 had three unique exons (Supplementary Figure S3). A ML phylogenetic tree of *O. dioica* and 14 other species in the family of Oleaceae suggested that *O. dioica* showed a close relationship with *O. brachiata* (Figure 3).

## Discussion and conclusions

In this study, the chloroplast genome sequence of *O. dioica* was constructed for the first time, and its genome structure was annotated. One hundred and thirty-one genes were found in the chloroplast genome of *O. dioica*. The chloroplast genome structure and genome size of *O. dioica* were similar to those of other published chloroplast genomes of *Olea* subgenus *Tetrapilus*, which has a genome size between 155,117 bp and 155,567 bp (Dong et al. 2022). However, variations were observed in the gene count of *O. dioica* compared to these species. Specifically, *O. dioica* exhibited one additional CDS and one tRNA gene. Phylogenetic analysis of 15 species of Oleaceae revealed that *O. dioica* is closely related to *O. brachiata*. The genus *Olea* includes three subgenera: *Olea*, *Paniculatae*, and *Tetrapilus*. In the phylogenetic tree, we found that the *Olea* subgenus *Tetrapilus* and *Olea* were well separated from each other, the characteristics of corolla tubes longer than corolla lobes and the absence of peltate scales were distinguish subgenus *Tetrapilus* from other subgenera in genus *Olea* (Besnard et al. 2002, 2009; Green 2002). We also found *O. caudatilimba* from the subgenus *Tetrapilus* appears to be a separate branch instead of gathering with the subgenus *Tetrapilus* clade, this result is similar to the recent study that *O. caudatilimba* was closely related to *Chionanthus mala-elengi* and *Chionanthus parkinsonii* (Dong et al. 2022; Xu et al. 2024). In the future, more extensive sampling will be conducted to see if any other species in subgenus *Tetrapilus* form a clade with *O. caudatilimba*. In conclusion, the chloroplast genome of *O. dioica* reported in this paper not only improves our understanding of the species but also contributes to the enhancement and integration of genomic data in Oleaceae.

## Supplementary Material

Supplemental Material

Supplemental Material

Supplemental Material

## Data Availability

The genome sequence data that support the findings of this study are openly available in GenBank of NCBI at https://www.ncbi.nlm.nih.gov/ under the accession number PP048999. The associated BioProject, SRA, and Bio-Sample numbers are PRJNA1061655, SRR27469441, and SAMN39276719, respectively.
