# Supplementary figures and images for: The complete chloroplast genome sequence of Olea dioica Roxb, 1820 (Oleaceae)
Source: Mitochondrial DNA B Resour. 2024 Jun 17;9(6):748–52. doi: 10.1080/23802359.2024.2366373 (PMC11185086; doi:10.1080/23802359.2024.2366373)

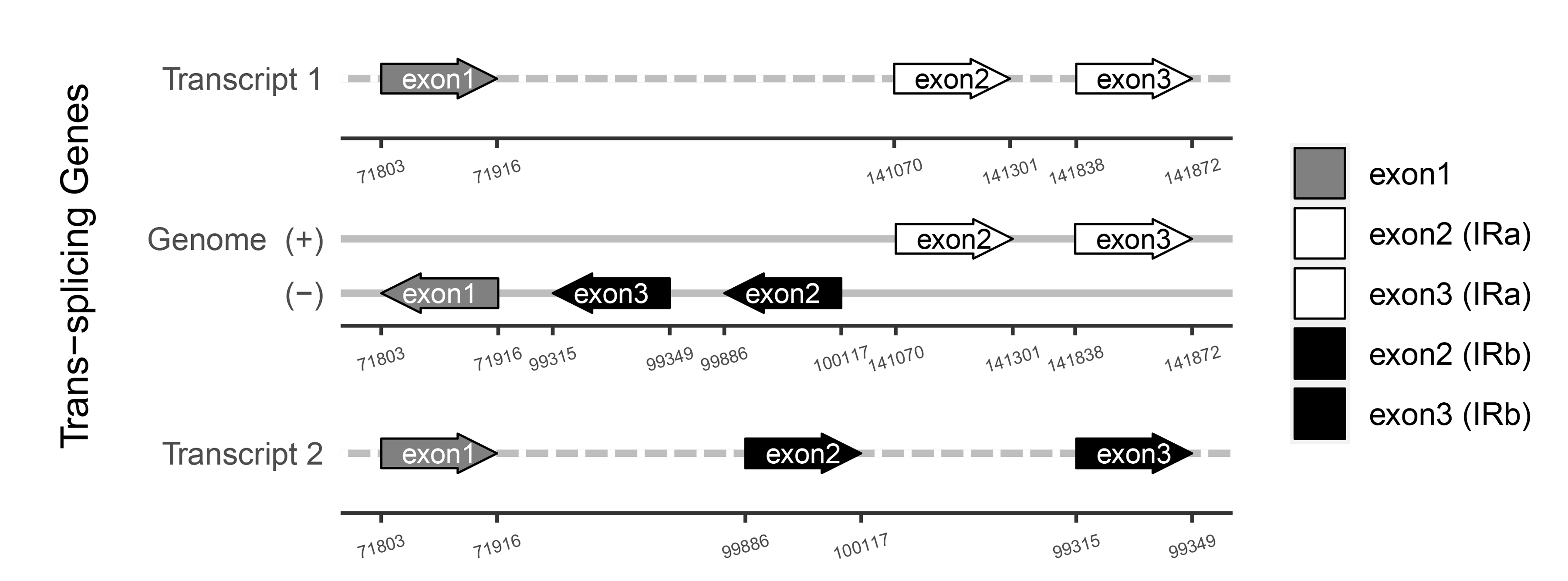

Supplement: Supplemental Material [file TMDN_A_2366373_SM0793.tif]

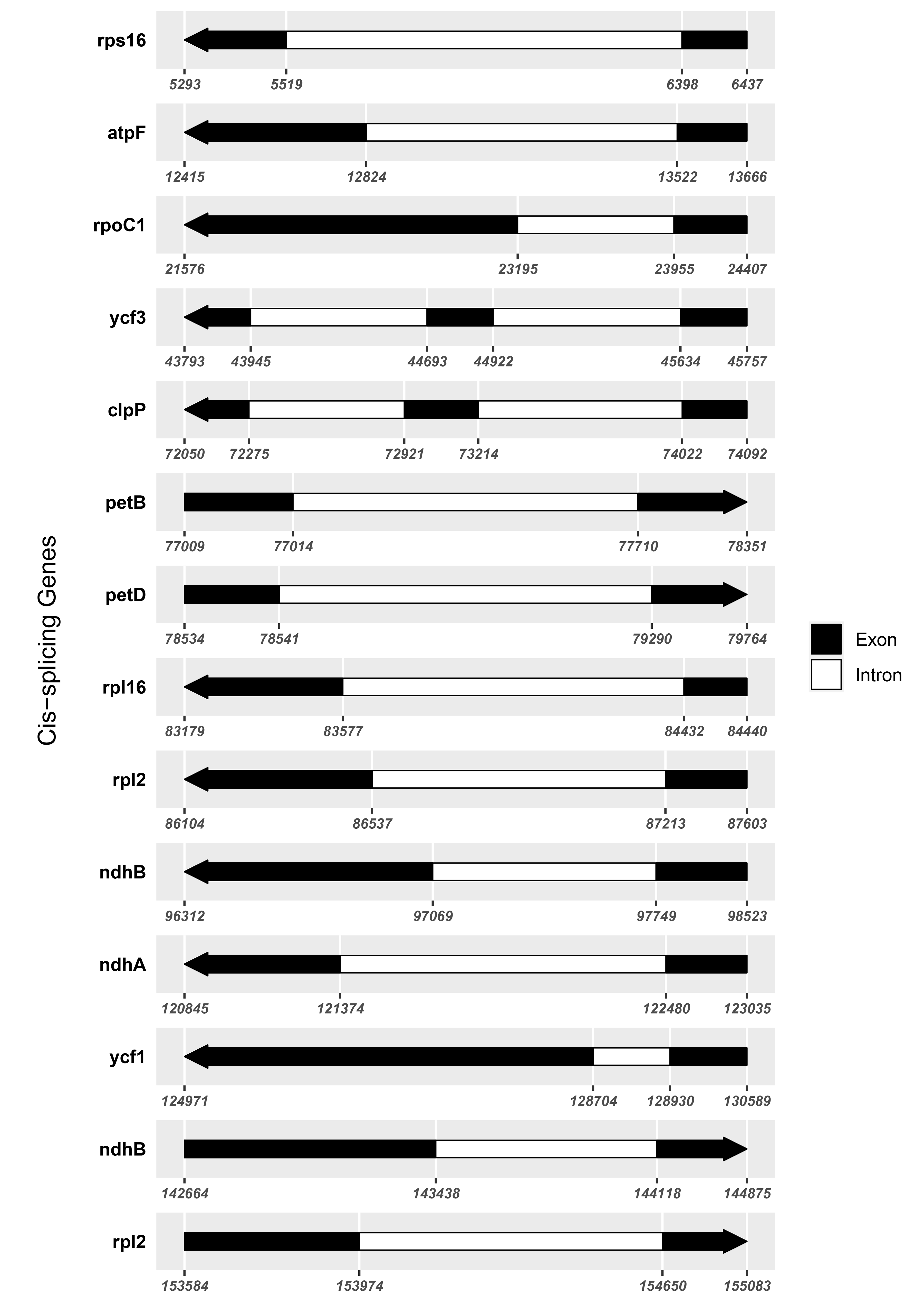

Supplement: Supplemental Material [file TMDN_A_2366373_SM0783.tif]

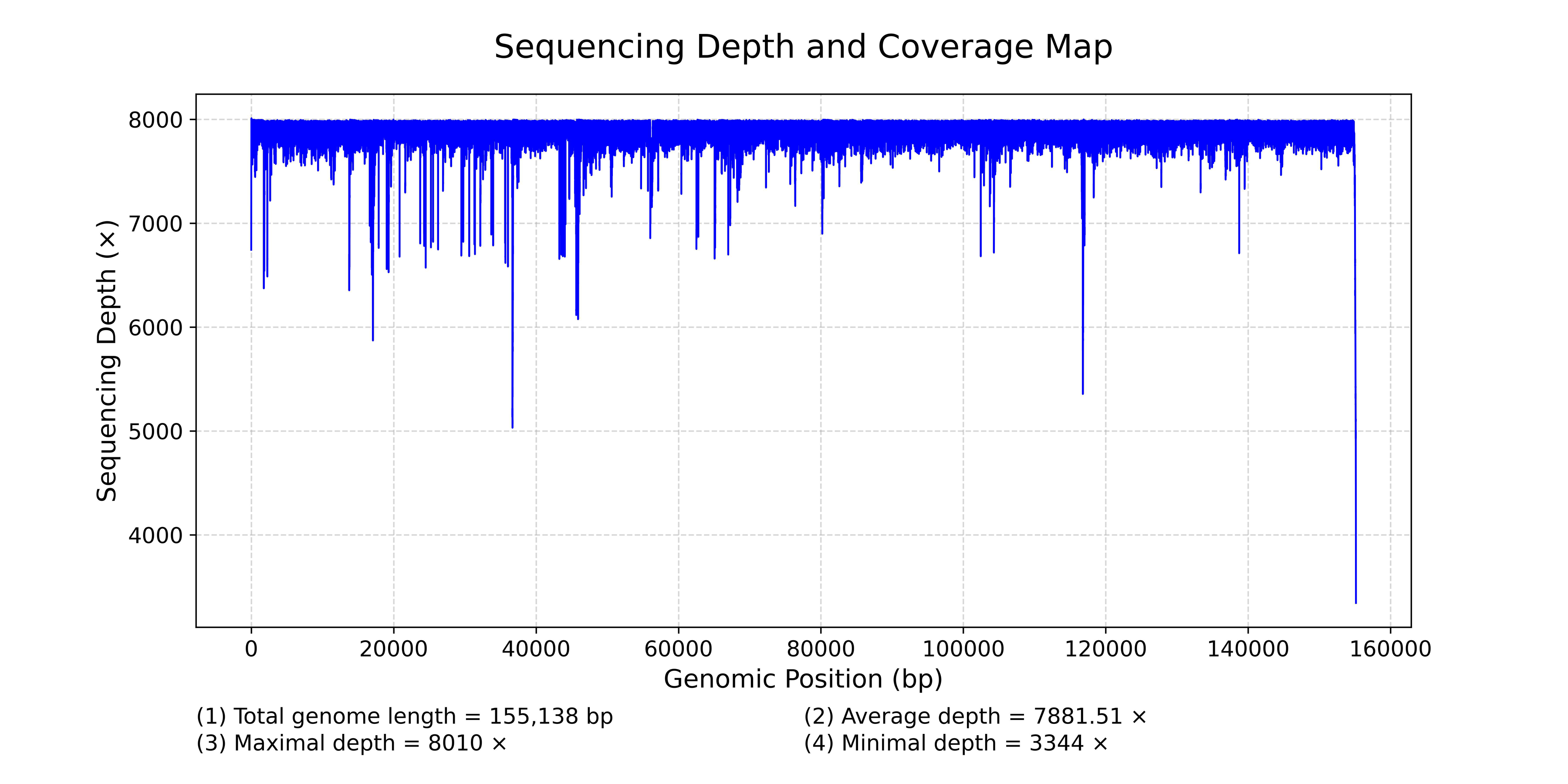

Supplement: Supplemental Material [file TMDN_A_2366373_SM0776.tif]
